# Dementia-related psychosis and the potential role for pimavanserin

**DOI:** 10.1017/S1092852920001765

**Published:** 2020-08-19

**Authors:** Jeffery L. Cummings, D. P. Devanand, Stephen M. Stahl

**Affiliations:** 1 Chambers-Grundy Center for Transformative Neuroscience, Department of Brain Health, School of Integrated Health Sciences, University of Nevada at Las Vegas (UNLV) and Cleveland Clinic, Lou Ruvo Center for Brain Health, Las Vegas, Nevada, USA; 2 Department of Psychiatry, Columbia University Medical Center, New York, New York, USA; 3 Department of Psychiatry, University of California, San Diego, La Jolla, California, USA

**Keywords:** Alzheimer’s, Parkinson’s, Vascular, Frontotemporal, Lewy

## Abstract

Dementia-related psychosis (DRP) is prevalent across dementias and typically manifests as delusions and/or hallucinations. The mechanisms underlying psychosis in dementia are unknown; however, neurobiological and pharmacological evidence has implicated multiple signaling pathways and brain regions. Despite differences in dementia pathology, the neurobiology underlying psychosis appears to involve dysregulation of a cortical and limbic pathway involving serotonergic, gamma-aminobutyric acid ergic, glutamatergic, and dopaminergic signaling. Thus, an imbalance in cortical and mesolimbic excitatory tone may drive symptoms of psychosis. Delusions and hallucinations may result from (1) hyperactivation of pyramidal neurons within the visual cortex, causing visual hallucinations and (2) hyperactivation of the mesolimbic pathway, causing both delusions and hallucinations. Modulation of the 5-HT_2A_ receptor may mitigate hyperactivity at both psychosis-associated pathways. Pimavanserin, an atypical antipsychotic, is a selective serotonin inverse agonist/antagonist at 5-HT_2A_ receptors. Pimavanserin may prove beneficial in treating the hallucinations and delusions of DRP without worsening cognitive or motor function.

## Introduction

No pharmacological agents are approved by the U.S. Food and Drug Administration (FDA) to treat dementia-related psychosis (DRP). Pimavanserin, an atypical antipsychotic that acts as a selective serotonin inverse agonist/antagonist at 5-HT_2A_ receptors (and to a lesser extent, at 5-HT_2c_ receptors), is the only FDA-approved treatment for hallucinations and delusions associated with Parkinson’s disease (PD) psychosis.^1,2^ A phase 2 study of pimavanserin in Alzheimer’s disease (AD) psychosis met its primary end point at week 6 (mean change in the Neuropsychiatric Inventory-Nursing Home version psychosis score of 3.76 points [SE 0.65] for pimavanserin and 1.93 points [SE 0.63] for placebo; *P* =0.045) with an acceptable tolerability profile and no worsening of cognition or motor function.^3^ Pimavanserin is being investigated (NCT03325556 and 2017-002227-13) for treating hallucinations and delusions associated with DRP across five common neurodegenerative dementias: AD dementia, PD dementia, dementia with Lewy bodies (DLB), frontotemporal dementia (FTD), and vascular dementia (VaD).^4–6^ This article discusses the emerging understanding of the neurobiology of psychosis, the current state of knowledge about the neurobiology of psychotic symptoms in dementia syndromes, and the hypothesized role of pimavanserin in treating DRP.

Approximately 7.9 million people in the United States have dementia, with this number projected to rise as the elderly population grows.^7–9^ Although AD dementia is most common in the United States (60%-80% of cases), other forms of dementia include PD dementia, DLB, FTD, and VaD. While distinctive features characterize each form of dementia, broad symptom overlap is observed.^10^

Dementia is a syndrome characterized by a decline in one or more cognitive domains (eg, memory, language, executive function, problem-solving, attention, and social cognition) sufficiently severe to compromise daily function.^11^ Besides the well-recognized cognitive deficits characteristic of dementia, noncognitive symptoms (often referred to as behavioral and psychological symptoms of dementia or neuropsychiatric symptoms [NPS]) are estimated to occur in up to 90% of individuals during the course of dementia. NPS include behavioral symptoms (such as agitation, aggression, disinhibition, elation, and irritability), aberrant motor behavior, anxiety, apathy, appetite changes, depression, sleep disturbances, and symptoms of psychosis.^12^

Although some dementia patients display no or only a few noncognitive symptoms, multiple NPS commonly occur simultaneously.^12^ The Agitation Definition Work Group of the International Psychogeriatric Association defines agitation as occurring in patients with a cognitive impairment or dementia syndrome; exhibiting behavior consistent with emotional distress; manifesting excessive motor activity, verbal aggression, or physical aggression; and evidencing behaviors that cause excess disability and are not solely attributable to another disorder.^13^ In contrast, DRP is defined by delusions or hallucinations occurring after the onset of cognitive decline, persisting for at least 1 month, and not better explained by delirium or some other mental illness.^11^ Both hallucinations and delusions are associated with behavioral symptoms such as physical and verbal aggression in patients with dementia.^14–16^

Prevalence estimates for psychosis range from 10% for FTD to 75% for DLB (Table 1).^16–30^ In the United States, an estimated 2.34 million people suffer from DRP.^16–30^ Visual hallucinations occur in all forms of dementia and are commonly observed in PD dementia and DLB.^31,32^ Delusions are also observed in all forms of dementia, most commonly paranoid delusions (eg, theft or spousal infidelity) and misidentifications, though the latter is sometimes considered a type of memory deficit rather than psychosis.^31,33^ DRP varies within and across patients in the psychotic symptoms manifested and in the severity of symptoms during the course of illness.^33^ DRP is more frequently observed in patients with more advanced dementia. DRP has consistently been associated with greater caregiver burden and more rapid progression to severe dementia, institutionalization, and death.^34–38^

Many forms of dementia, aside from VaD, result from neurodegenerative disease and are associated with various proteinopathies characterized by protein misfolding and aggregation. Aberrant aggregated proteins in AD produce β-amyloid plaques and neurofibrillary tangles (hyperphosphorylated tau aggregates).^39,40^ In DLB and PD dementia, Lewy bodies and Lewy neurites, composed primarily of phosphorylated α-synuclein aggregates, accumulate preferentially in limbic brain regions.^41–43^ FTD may be associated with either aggregated tau protein or aggregated trans-activator regulatory DNA-binding 43 (TDP-43) protein.^44^

Post-mortem analyses have revealed most dementia patients exhibit mixed pathology comprising abnormal protein aggregates plus vascular changes.^45,46^ In one study of community-dwelling adults, 56% of dementia patients were diagnosed with multiple underlying pathologies (AD in combination with either PD/DLB, infarctions representing vascular brain injury, or both).^46^ After adjusting for age, individuals with multiple diagnoses were deemed to be nearly three times more likely to develop dementia as those with a single underlying pathology.^46^ In a separate study, database analyses revealed that 59% to 68% of patients with AD neuropathology also displayed Lewy body pathology or vascular brain injury.^45^

## Neurobiology of Psychosis

The underlying mechanisms behind psychosis in dementia are unknown. The neurobiology of psychosis has primarily been examined in studies of patients with schizophrenia and animals investigated in pharmacological probe paradigms.^47^ Taken together, the evidence suggests alterations in multiple signaling pathways— including dopaminergic, gamma-aminobutyric acid (GABA)ergic, glutamatergic, and serotonergic neurotransmission—may contribute to psychosis.^48^ Excess dopamine signaling in the mesolimbic pathway, which projects from the ventral tegmental area (VTA) to the nucleus accumbens, has been demonstrated to promote positive symptoms, primarily delusions and hallucinations.^49^ Under basal conditions, GABAergic interneurons provide inhibitory regulation of the activity of cortical pyramidal neurons. Inhibition of N-methyl-D-aspartate (NMDA) receptors reduces the inhibitory activity of GABAergic interneurons, resulting in glutamatergic hyperfunction. In rodent models, NMDA receptor antagonism or cortical and hippocampal NMDA receptor deletion results in psychotic-like behaviors.^50,51^ Similarly, ketamine-induced NMDA antagonism significantly increases positive symptoms in haloperidol-treated patients diagnosed with schizophrenia.^52^ Glutamatergic projections from the prefrontal cortex provide tonic control of dopaminergic neurons in the VTA. Microdialysis and electrical stimulation rodent models reveal that glutamatergic hyperfunction increases burst firing of dopaminergic neurons in the VTA, stimulating the mesolimbic pathway.^53,54^

These findings are supported by basic pharmacological intervention studies showing that multiple signaling pathways contribute to psychosis. These observations include dopamine D2 receptor activation via psychostimulants, glutamate NMDA receptor inhibition via dissociative anesthetics, and serotonin 5-HT_2A_ receptor activation via psychedelics, all of which have been reported to precipitate psychotic symptoms.^55^ Methamphetamine, which is a substrate for the dopamine transporter (DAT), increases the firing rate of cultured rat dopamine neurons, triggering an excitatory response at dopamine concentrations lower than those required for D2 autoreceptor activation.^56^ Both methamphetamine and the DAT inhibitor cocaine, which increases extracellular dopamine levels via uptake inhibition, induce paranoid delusions as well as auditory and tactile hallucinations in stimulant-dependent individuals.^57,58^ Ketamine, a noncompetitive NMDA receptor antagonist, significantly increases hallucinations and delusions in schizophrenic patients following administration of subanesthetic doses and increases regional cerebral blood flow to the anterior cingulate cortex, while decreasing blood flow to the hippocampus and primary visual cortex.^59,60^ In addition, subanesthetic doses of ketamine induces acute auditory verbal hallucinations and paranoid delusions in control subjects without psychosis in a reduced-stimulation environment.^61^ Administration of the serotonin 5-HT_2A_ receptor agonist lysergic acid diethylamide (LSD) to healthy volunteers results in increased cerebral blood flow and enhanced resting-state functional connectivity in the visual cortex, which strongly correlates with visual hallucinations.^62^ Administration of the 5-HT_2A_ receptor agonist psilocybin to hallucinogen-naïve adults precipitates mystical delusions that persist at 14-month follow-up in 60% of subjects.^63^ In a separate study in healthy human volunteers, psychotic symptoms that closely mimicked those observed in first-episode schizophrenic patients (including sensory misperceptions and thought-process disruption) occur within 20 to 30 minutes of psilocybin administration. Pretreatment with the 5-HT_2A_ receptor antagonist ketanserin inhibits psilocybin-induced psychosis in a dose-dependent manner, while pretreatment with the D2 receptor antagonist haloperidol does not affect psilocybin-induced hallucinations, further supporting the idea that certain symptoms of hallucinogen-induced psychosis (such as visual hallucinations) result from 5-HT_2A_ agonism, while others (such as auditory hallucinations) are more strongly linked to the D2 receptor.^64^ Additional support for multifactorial signaling stems from the observation that conventional antipsychotics exert their effects primarily via D2 receptor inhibition, while atypical antipsychotics act as serotonin–dopamine antagonists, inhibiting both D2 and 5-HT_2A_ receptors.^65^

**Figure 1.**
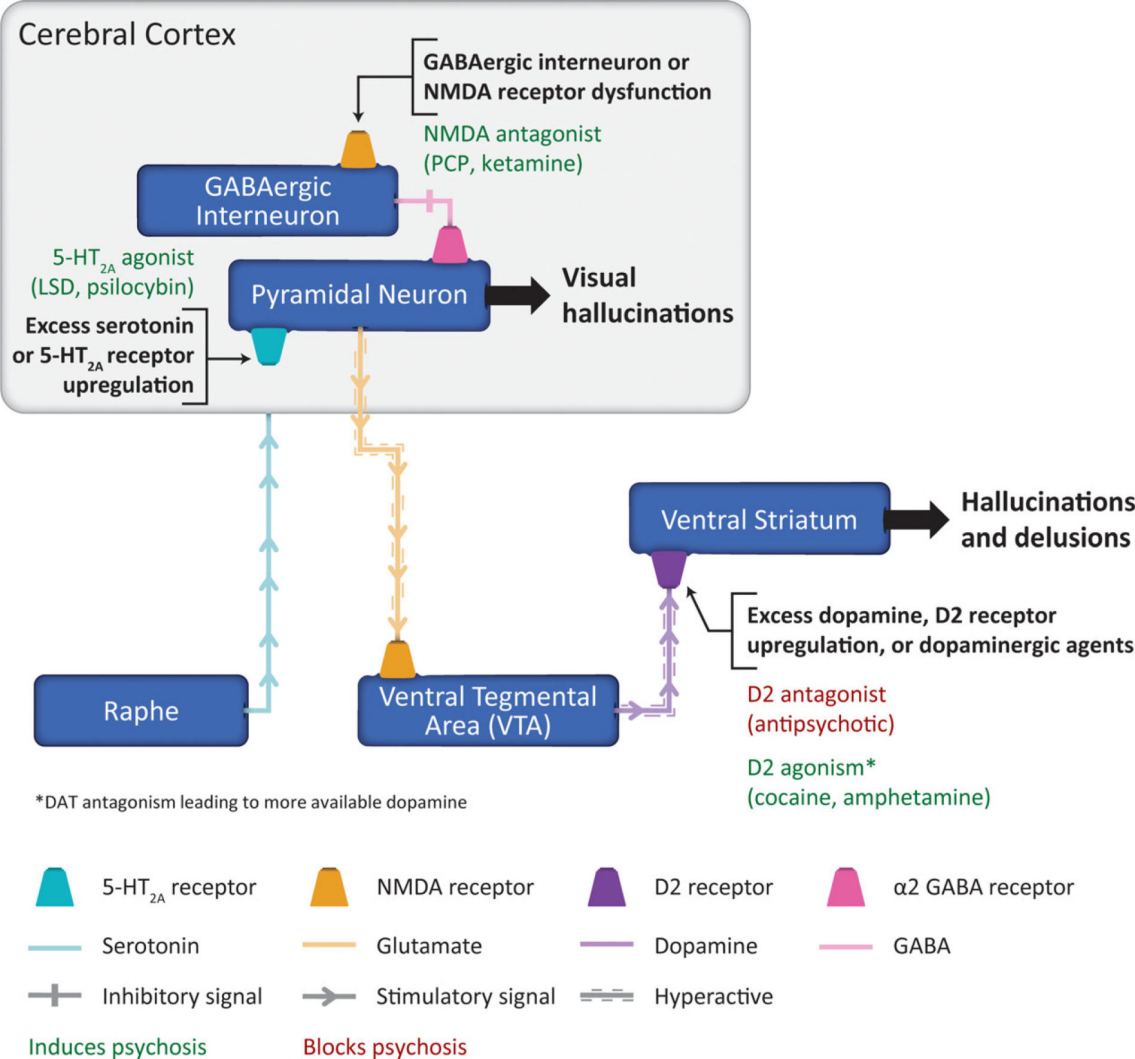
Hypothesized cortical–limbic psychosis pathway and proposed mechanism of disease for DRP. Neurobiological and pharmacological evidence suggests that hallucinations and delusions are precipitated by overactivation of the mesolimbic pathway, while visual hallucinations are mediated via overactivation of the visual cortex. Dissociative anesthetic-induced (ie, PCP, ketamine) glutamate NMDA receptor antagonism, psychedelic-induced (ie, LSD, psilocybin) serotonin 5-HT_2A_ receptor agonism, and psychostimulant-induced (ie, amphetamine, cocaine) dopamine D2 receptor agonism/DAT antagonism have all been reported to precipitate hallucinations and delusions. In contrast, antipsychotic-mediated D2 and 5-HT_2A_ antagonism treat both hallucinations and delusions. GABAergic interneuron or NMDA receptor dysfunction, and excess serotonin or 5-HT_2A_ receptor upregulation in the cerebral cortex can result in sustained activation of pyramidal neurons and may lead to hyperactive glutamatergic signaling to the VTA, resulting in excess dopamine or D2 receptor upregulation in the ventral striatum, triggering hallucinations and delusions in DRP.^55–56,70–87^

Taken together, neurobiological and pharmacological evidence points to a common, interconnected cortical–limbic psychosis pathway (Figure 1). Both the occurrence of hallucinogen 5-HT_2A_ receptor effects in the prefrontal and visual cortices, and the observation that loss of serotonin nerve terminals in the prefrontal and visual cortices of patients with PD psychosis leads to upregulation of 5-HT_2A_ receptors in the cortex support this hypothesis. A possible convergence of many of these pathways into 5-HT_2A_ modulated systems is suggested by the observation that the 5-HT_2A_ receptor is affected by essentially all atypical antipsychotics, which led to the development of pimavanserin, a 5-HT_2A_-selective inverse agonist/antagonist.^66^ In preclinical rodent studies, pimavanserin reduced both amphetamine- and NMDA receptor antagonist-induced hyperactivity when combined with haloperidol or haloperidol and risperidone, respectively.^67^ Additionally, pimavanserin was shown to reverse psychosis-like behaviors in rodent models of PD psychosis without impairing motor performance or interfering with the efficacy of PD medications.^68^ Finally, pimavanserin increased dopamine release in the medial prefrontal cortex but not in the nucleus accumbens, suggesting pimavanserin may be beneficial for cognitive, negative, and psychotic symptoms.^69^

## Changes in Dementia that may Result in Psychosis Pathway Dysfunction

The cortical–limbic psychosis pathway provides numerous points where dysfunction could trigger delusions and/or hallucinations. The pathology and dysfunction across the dementias leading to delusions and/or hallucinations may be different in specific dementias but each is positioned to affect the function of the cortical–limbic system thought to mediate psychosis. The evidence for dysfunction which could disrupt the cortical–limbic psychosis pathway in each dementia is described below.

### PD dementia

While dopamine depletion in the dorsal striatum due to loss of nigrostriatal neurons results in the characteristic motor symptoms of PD, serotonin dysfunction is thought to be the underlying cause of PD psychosis. Post-mortem analyses indicate Lewy body and Lewy neurite deposition in the raphe nucleus magnus, obscurus, and pallidus, and the gigantocellular nucleus of the medullary lateral reticular formation nuclei (which comprise the serotonergic caudal brainstem complex) precedes deposition in dopaminergic midbrain neurons.^88^ In addition, significant decreases in serotonin, the serotonin transporter (SERT), and the serotonin metabolite 5-hydroxyindoleacetic acid (5-HIAA) are observed in the caudate in post-mortem analyses of PD patients.^72^ During the early stages of PD, SERT expression is diminished in the forebrain, but preserved in the caudate, and increased serotonergic uptake is observed in the thalamus and raphe nuclei, indicating a shift toward mesolimbic and mesocortical dysfunction.^73,74^ As PD progresses, SERT expression in the caudate decreases and is correlated with disease stage.^75^ In PD patients with visual hallucinations, upregulation of 5-HT_2A_ receptors has been observed using positron emission tomography in the inferolateral temporal cortex—in complex visual processing—as well as other portions of the ventral visual pathway, including the bilateral inferior occipital gyrus and right fusiform gyrus.^76,77^ 5-HT_2A_ receptor upregulation may represent a compensatory mechanism by which the brain attempts to counteract reductions in serotonin levels.^77^ In a double-blind, randomized, placebo-controlled phase 3 study of the efficacy of pimavanserin in the treatment of hallucinations and delusions associated with PD psychosis, a post-hoc subgroup analysis revealed patients with cognitive impairment (Mini-Mental State Examination scores 21–24) demonstrate a significantly greater improvement in Scale for the Assessment of Positive Symptoms-PD scores from baseline to week 6 with pimavanserin as compared to placebo-treated patients (*P* =0.002), suggesting pimavanserin may exhibit a more robust effect in cognitively impaired patients.^89^

Imaging studies demonstrate that PD dementia is associated with more widespread cortical cholinergic depletion than that observed in patients with PD who do not exhibit dementia.^90,91^ Modulation of cholinergic activity has been reported to decrease hallucinations and delusions in PD dementia patients. Treatment with the acetylcholinesterase inhibitor donepezil significantly reduces hallucinations and paranoid ideation, as well as overall rating scale scores for PD psychosis.^92^ At present, the underlying mechanism for these effects is unknown. Serotonin has been reported to inhibit acetylcholine release from cortical cholinergic nerve terminals^93^ and 5-HT receptor inverse agonism/antagonism may represent another therapeutic strategy to modulate cortical cholinergic activity.

### Dementia with Lewy bodies

In DLB, the density of Lewy bodies in limbic areas (highest density in the amygdala) is significantly higher than in neocortical areas.^94^ As in PD, serotonin dysfunction is thought to be the underlying cause of psychosis in DLB. Cortical 5-HT_2_ binding differs between patients with DLB with and without hallucinations. Significant deficits in 5-HT_2_ binding are observed in cortical layers III and V (deep cortical layers that contain pyramidal neurons) in patients who did not experience hallucinations, whereas a 5-HT_2_ binding deficit was observed only in one upper cortical layer in patients who did experience hallucinations, suggesting 5-HT_2_ receptor preservation in the temporal cortex may contribute to hallucinations in this population.^78^ This hypothesis is further supported by the observation that serotonergic receptor binding and 5-HIAA levels were significantly decreased in nonhallucinating vs hallucinating patients with DLB in a neurochemical analysis of the temporal cortex. In the same study, downregulation of choline acetyltransferase activity was observed in the temporal and parietal cortices, particularly in those experiencing hallucinations.^79^ Treatment with the cholinesterase inhibitor rivastigmine decreased delusions and hallucinations in patients with DLB compared to placebo.^95^ The mechanism underlying the efficacy of cholinesterase inhibition in the treatment of DLB remains unresolved. However, hallucinations in patients with DLB have been proposed to result from imbalances between the serotonergic and cholinergic inputs to the cortex, suggesting restoration of the balance between the two inputs may account for this effect.^96^

### AD dementia

Although serotonergic signaling is altered in AD, it may not be the primary dysfunction contributing to psychosis. When compared to healthy controls, significant reductions in 5-HT_2_ receptor binding and expression have been observed in the frontal, temporal, and cingulate cortices, as well as the amygdala and hippocampus, in patients with AD.^97–101^ In a study of psychopathology in late-onset AD patients, the 5-HT_2A_ receptor polymorphism 102-T/C was significantly associated with visual and auditory hallucinations, and the 5-HT_2C_ receptor polymorphism Cys23Ser was significantly associated with visual hallucinations, implicating neurodegeneration in the biology of the psychotic symptoms of individuals expressing these genetic variations. However, the 5-HT_2A_ 102-T/C polymorphism reduces 5-HT_2A_ receptor expression while the 5-HT_2C_ Cys23Ser polymorphism leads to functional downregulation of the 5-HT_2C_ receptor.^102–104^

Concomitant serotonergic and cholinergic deficits have been observed in the frontal and temporal cortices in AD patients as compared to healthy controls, and the ratio of serotonin to acetylcholinesterase in the temporal cortex is correlated with psychosis in female patients.^105^ Cholinesterase inhibitors used in the treatment of AD may improve hallucinations and delusions in some patients.^106^

There is evidence of GABAergic and glutamatergic dysfunction in AD which might disrupt the cortical–limbic psychosis pathway. Significant reductions in GABA concentrations in numerous cortical areas, including the temporal, frontal (orbitofrontal and premotor cortex), parietal, and occipital cortices, are observed in biopsy and autopsy specimens obtained from AD patients.^107–111^ Alterations in NMDA-mediated glutamatergic signaling appears to vary throughout the course of AD. Under normal circumstances, glutamate regulates the inhibitory tone of these GABA neurons; amyloid appears to increase the sensitivity of these receptors to glutamate, leading to glutamatergic hyperactivation and GABAergic neuronal degeneration. During the later stages of disease progression, excessive GABAergic neuronal degeneration results in NMDA receptor hypofunction.^80^ Evidence of GABAergic or glutamatergic dysfunction has yet to be directly associated with psychosis in AD. Treatment of AD patients with the NMDA receptor antagonist memantine, which was shown to inhibit the excitotoxic effects of NMDA glutamate receptors, has occasionally been reported to worsen or induce new visual hallucinations in AD patients.^81^

### Frontotemporal dementia

Deficiencies in the serotonergic system have been reported in imaging studies, post-mortem tissue analyses, and cerebrospinal fluid studies of patients with FTD. Decreased 5-HT_2A_ receptor expression has been observed in the orbitofrontal, frontal medial, and cingulate cortices of such patients.^82^ Data regarding how GABAergic and glutamatergic signaling are affected in FTD are incomplete; however, loss of glutamatergic pyramidal cells and GABAergic neurons in the upper layers of the frontal and temporal cortices has been reported.^83^ MRI studies have revealed widespread limbic atrophy in FTD,^112,113^ suggesting disruption of the cortical–limbic psychosis pathway may occur. However, data regarding dysfunction specific to psychosis in this population are not yet reported, possibly because psychosis is less common in FTD.

### Vascular dementia

Serotonergic dysfunction is present in VaD, with increased 5-HT_1A_ and 5-HT_2A_ receptor binding observed in the temporal cortex of post-mortem tissue samples from multi-infarct VaD patients.^84^ The relationship between this increased receptor binding and psychosis is unknown. Data regarding the roles of GABAergic and glutamatergic signaling in VaD is lacking.

### Neurotransmission alterations across dementias

While the strength of the evidence varies across these dementias, all have been associated with alterations in neurotransmission which have the potential to impact the cortical–limbic psychosis pathway. Each of these conditions is highly heterogeneous and the likelihood an individual patient will develop psychosis varies greatly. Further research is required to confirm how etiological factors, such as the anatomical locations of neurodegeneration, compensatory mechanisms, and genetic influences contribute to the development of psychosis.

## Proposed Mechanism for DRP

The proposed mechanism for DRP is believed to involve a common cortical–limbic psychosis pathway (Figure 1). Cortical GABAergic interneuron or NMDA receptor dysfunction is hypothesized to result in loss of inhibitory tone, leading to hyperactivity of glutamatergic neurons that signal to the VTA. Alternatively, excessive signaling via 5-HT_2A_ receptors on pyramidal glutamate neurons can lead to hyperactive glutamatergic neurons that signal to the VTA. Sustained hyperactive glutamatergic signaling then leads to mesolimbic dopamine pathway hyperactivation, resulting in hallucinations and delusions. Excess signaling via 5-HT_2A_ receptors in the visual cortex may be responsible specifically for visual hallucinations.^64–66,70,72–87^

The cortical–limbic psychosis pathway can be triggered in a variety of different ways across dementias based on which neurons are lost or damaged and which signaling pathways become disordered but appears to be responsive to serotonin modulation. Cortical GABA interneuron dysfunction, excess serotonin, cortical 5-HT_2A_ receptor upregulation, excess striatal dopamine, striatal D2 receptor upregulation, and excess glutamate signaling all have the potential to contribute to pathway dysfunction.

## Proposed Mechanism of Action of Pimavanserin

**Figure 2.**
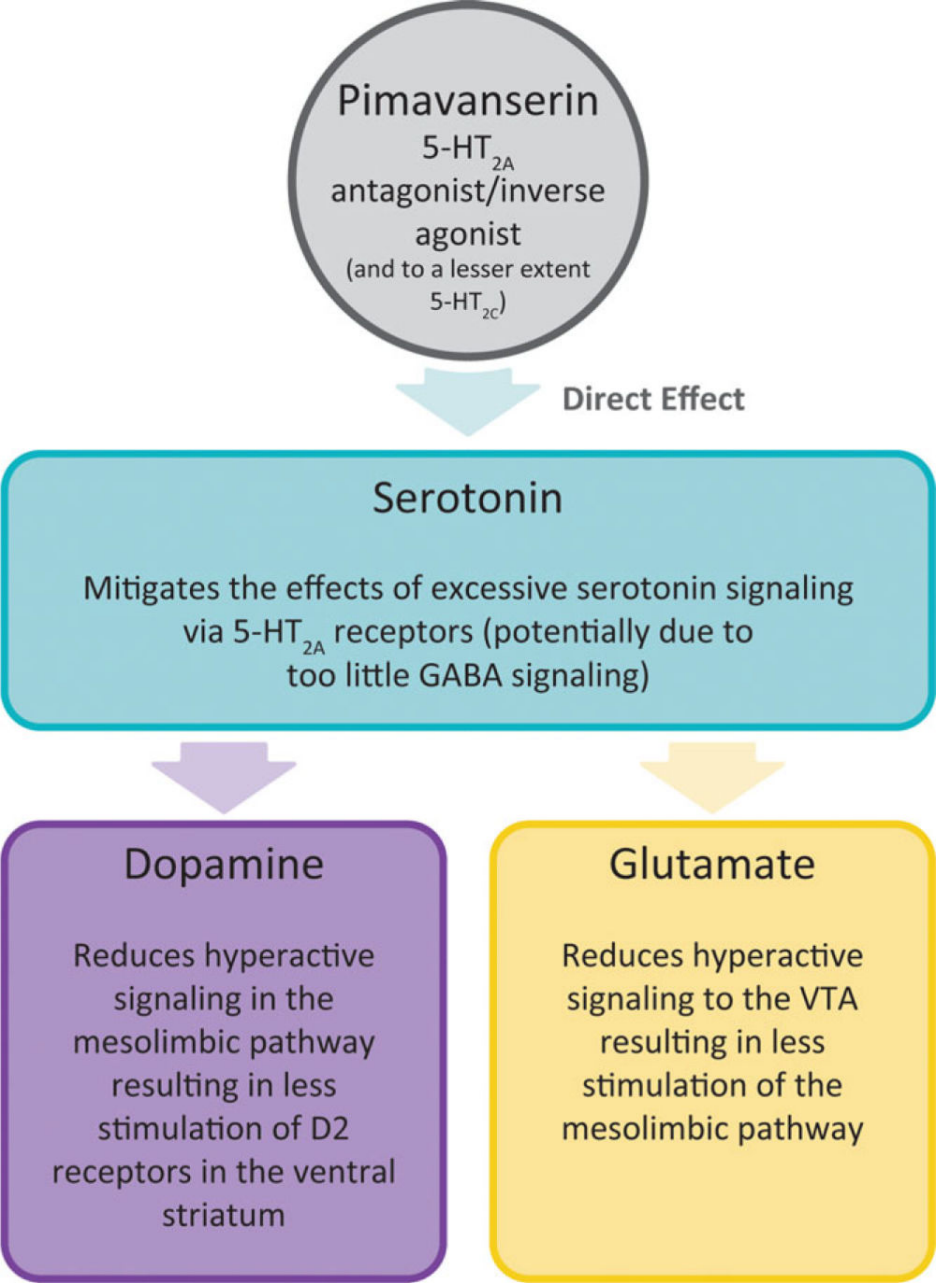
Pimavanserin-mediated 5-HT_2A_ receptor inhibition: hypothesized modulation of signaling through a variety of neurotransmitters. Through 5-HT_2A_ antagonism/reverse agonism, pimavanserin is proposed to act as a targeted serotonergic modulator of circuits, mitigating the effects of GABAergic deficits and excess serotonergic signaling, while also reducing hyperactive glutamatergic signaling and mesolimbic pathway activation.

Across the underlying causes of cortical–limbic psychosis pathway hyperactivation, 5-HT_2A_ receptor antagonism represents a common point of regulation and for treatment intervention with antipsychotics.^66^ Pimavanserin is a selective serotonin inverse agonist/antagonist at 5-HT_2A_ receptors, with 40-fold less activity at 5-HT_2C_ receptors and no affinity for dopaminergic, histaminergic, muscarinic, or adrenergic receptors, and is proposed to act as a targeted serotonergic modulator of circuits (Figure 2).^66^ Pimavanserin is thought to reduce the activity of these receptors to below basal levels and regulate the effects of both cortical GABAergic deficits and excess cortical serotonergic signaling. This is posited to decrease visual hallucinations and attenuate glutamate signaling to the VTA and mesolimbic pathway, further decreasing delusions and hallucinations.

## Summary

DRP is a commonly occurring phenomenon across dementias, yet no pharmacological agents are currently approved by the FDA for DRP treatment. Commonly used typical and atypical antipsychotics have an increased risk of death and other treatment-limiting side effects.^114,115^ While the etiology of DRP is unknown, neurobiological and pharmacological evidence supports a common, interconnected cortical–limbic psychosis pathway mediating DRP, which may be modified via 5-HT_2A_ receptor inverse agonism/antagonism. The only FDA-approved agent to treat hallucinations and delusions associated with PD psychosis, pimavanserin (proposed to act as a targeted serotonergic modulator of circuits), is being investigated to treat DRP in a phase 3 trial completed in 2019.^5^ The selectivity of pimavanserin presents a potential novel mechanism for the management of hallucinations and delusions associated with DRP.^66,89^

## Figures and Tables

**Table 1.** Prevalence ranges for psychosis, delusions, and hallucinations in AD dementia, VaD, DLB, PD Dementia, and FTD

	Alzheimer’s Disease Dementia	Vascular Dementia	Dementia with Lewy Bodies	Parkinson’s Disease Dementia	Frontotemporal Dementia
Overall psychosis prevalence	30%	15%	75%	50%	10%
Delusions prevalence	10%–39%	14%–27%	40%–57%	28%–50%	2.3%–6%
Hallucinations prevalence	11%–17%	5%–14%	55%–78%	32%–63%	1.2%–13%
